# Systematic review of the training process of minimally invasive surgery in neonates and infants

**DOI:** 10.1007/s00383-025-06095-7

**Published:** 2025-06-26

**Authors:** Petra Zahradníková, Jozef Babala, Martin Lindák, Rebeka Pechanová, Silvia Hnilicová, Marián Molnár, Barbora Nedomová, Lucie Poš, Vojtěch Dotlačil, Barbora Kučerová

**Affiliations:** 1 Department of Pediatric Surgery, Faculty of Medicine, Comenius University and National Institute of Children’s Diseases, Bratislava, Slovakia; 2 Institute of Medical Education and Simulations, Faculty of Medicine, Comenius University, Bratislava, Slovakia; 3 Department of Pediatric Surgery, Jessenius Faculty of Medicine, Comenius University, Martin, Slovakia; 4 Department of Pediatric Anesthesiology and Intensive Medicine, Faculty of Medicine, Comenius University and National Institute of Children’s Diseases, Bratislava, Slovakia; 5 Department of Pediatric Surgery, Charles University and Motol University Hospital Prague, Prague, Czech Republic

**Keywords:** Simulation models, Minimally invasive surgery, (MIS) in neonates and infants, Laparoscopic surgery, Thoracoscopic surgery, Simulation-based training

## Abstract

**Purpose:**

Minimally invasive surgery (MIS) in neonates and infants presents technical challenges and is still unfamiliar to many paediatrics surgeons. This study aims to identify currently available simulators for neonatal/infant MIS training, to assess their validity, level of evidence, and related recommendations.

**Methods:**

The review followed PRISMA guidelines and was registered in PROSPERO (CRD420250581050). Electronic search limited to English articles was performed through PubMed/MEDLINE, SCOPUS, Web of Science and Cochrane Database from January 2010 to June 2024.

**Results:**

Out of 1084 identified records, 72 studies met the inclusion criteria and were analysed across general, gastrointestinal, thoracic, and urological MIS specialties. Recent efforts have led to the development of 3D-printed, animal-based, and hybrid models several of which showed high fidelity, skill differentiation, and educational value. Despite promising results, no universal MIS training model exists for neonate/infant patients, highlighting the need for structured, proficiency-based curricula. Overall, studies demonstrated moderate levels of evidence and recommendation, supporting integration of cost-effective simulation into paediatrics MIS training

**Conclusion:**

This systematic review highlights the need for validated, standardized simulation models and proficiency-based curricula to optimize neonate and infant MIS training and guide future research toward improving model fidelity, accessibility, and long-term educational outcomes.

## Introduction

Minimally invasive techniques are being adopted as the gold standard around the world for an ever-growing number of indications in pediatrics surgery. Therefore, the need for well-trained and certified laparoscopic surgeons is expected to increase [1]. Minimally invasive surgery (MIS) is technically more difficult than traditionally open surgery and several complications can happen at the beginning of practice [2]. The learning curve for MIS is much longer than for open surgery [3]. Performing MIS in neonates and infants thus poses technical challenges and remains an unfamiliar procedure for numerous pediatrics surgeons. Furthermore, the majority of infants undergoing MIS repair are exceptionally small at the time of surgery, resulting in a confined and narrow surgical field [4]. On the other hand, training of pediatrics surgeons faces many challenges nowadays. Decreased birth rates, reduced numbers of newborns with congenital surgical conditions, and regulation of working hours have reduced exposure to surgical opportunities for surgeons in training. In other words, gone are the days of the “see one, do one, teach one” philosophy in the operating room [5, 6]. Nowadays, besides reduced practical exposure, the traditional form of training is fast becoming obsolete due to the increased expectations of patient safety, surgeon performance, and transparency. Simulation has proven to be a fundamental tool in surgical education and can serve as a solution [7, 8]. In recent years, shortening of the learning curve has been demonstrated by implementing of wide range of surgical models [9]. Thanks to the repeatability of the simulations, both surgeons and residents have the great opportunity to learn surgical techniques or particular steps with the possibility of making mistakes, practice abilities, and procedures in standardized and supervised situations. Although simulation training in surgery can never completely replace clinical exposure, it can offer many of the necessary skills. Simulation-based training (SBT) in pediatrics surgery is still in its early stages. While simulations have already been commonly adopted in other surgical specialties, such as urology, orthopedics, and otorhinolaryngology, pediatrics surgery lags behind general surgery in the field of simulations [10]. There are currently many more simulators designed for adult anatomy than for children or newborns. In simulations, the common practice is to strive for a high level of fidelity to enhance the realism of the experience [10]. The level of fidelity can range from low to high, with the optimal choice depending on educational objectives. Spatial constraints during MIS in pediatrics operations make the techniques more challenging compared to adult patients. The aim of this review is to identify the current SBT models for MIS training in infants and neonates described in the literature, evaluate their validity, level of evidence (LoE), and level of recommendations (LoR).

## Methodology

This study was conducted following the Preferred Reporting Items for Systematic Reviews and Meta-Analysis (PRISMA) guidelines and statement [12]. The protocol for this systematic review was registered with PROSPERO (registration number CRD420250581050).

## Search strategy and study selection

An electronic search was performed using the PubMed/MEDLINE, SCOPUS, Web of Science and Cochrane Database from January 2010 to June 2024. The search was limited to English language articles. We considered all studies published as full papers in English, including randomized controlled trials (RCTs), prospective observational studies, case–control studies and case series. We excluded case reports, studies not published as full papers (e.g., conference abstracts) and studies published in languages other than English. The literature search was performed by seven reviewers (PZ, BK, LP, VD, MM, BN and ML). Review Question: This systematic review aims to evaluate the impact of simulation-based surgical training on clinically relevant outcomes in minimally invasive procedures performed in neonates and infants.

According to the PICOS format, the following terms were used to select the articles retrieved from the literature search: P (population): pediatric/pediatric surgeon, surgeon, resident, trainee. I (intervention): minimally invasive surgery, MIS, surgical training, curriculum, simulation training. C (comparison): no comparison. O (outcomes): surgeon’s learning curve on any outcome, such as operative time or postoperative outcomes/complications, simulator validation. S (study design): due to the expected paucity of studies on the topic in the literature, all types of study design were considered, except case report, systematic review, training models for open surgery, surgical subspecialty was not pediatric surgery. The search protocols included the following electronic keywords terms: infant, neonate OR infant AND surgical procedures, operative AND simulation training AND minimally invasive surgical procedures OR laparoscopy OR thoracoscopy AND simulation training.

## Data extraction

All articles identified through the search strategy underwent a multi-step processing by members of the systematic review team. First, duplicates were removed (by P.Z.). Titles were independently screened by two co-authors per database, with discrepancies resolved based on abstract review. Abstracts were then independently screened by two reviewers, with any disagreements resolved by full-text assessment. Full-text screening was performed by all co-authors, and any conflicts were resolved through discussion until consensus was reached. A reference check of included studies was used for supplementary identification of relevant articles. Articles were included if they described or validated neonate/infant MIS simulators, or pediatric simulators applicable to neonatal procedures or anatomical size. Studies were excluded if they were systematic reviews, case reports, open surgery training models, or focused on non-pediatric subspecialties. For each included article, data were extracted using a structured Excel sheet, including first author, year of publication, number of participants/experts, type of simulator validation, level of evidence (LoE), and level of recommendation (LoR). Models were categorized by procedure type: neonate/infant general surgery, congenital diaphragmatic hernia (CDH), esophageal atresia with tracheoesophageal fistula (EA/TEF), upper GI, hepatobiliary, thoracic, and urological procedures. The modified Oxford Centre for Evidence-Based Medicine (OCEBM) classification was used to assess LoE and LoR ((https://www.cebm.ox.ac.uk/resources/levels-of-evidence/ocebm-levels-of-evidence). Risk of bias was evaluated using the Prediction Model Risk of Bias Assessment Tool (PROBAST), covering participants, predictors, outcomes, and analysis [13].

## Results

### Description of studies

**Fig. 1:**
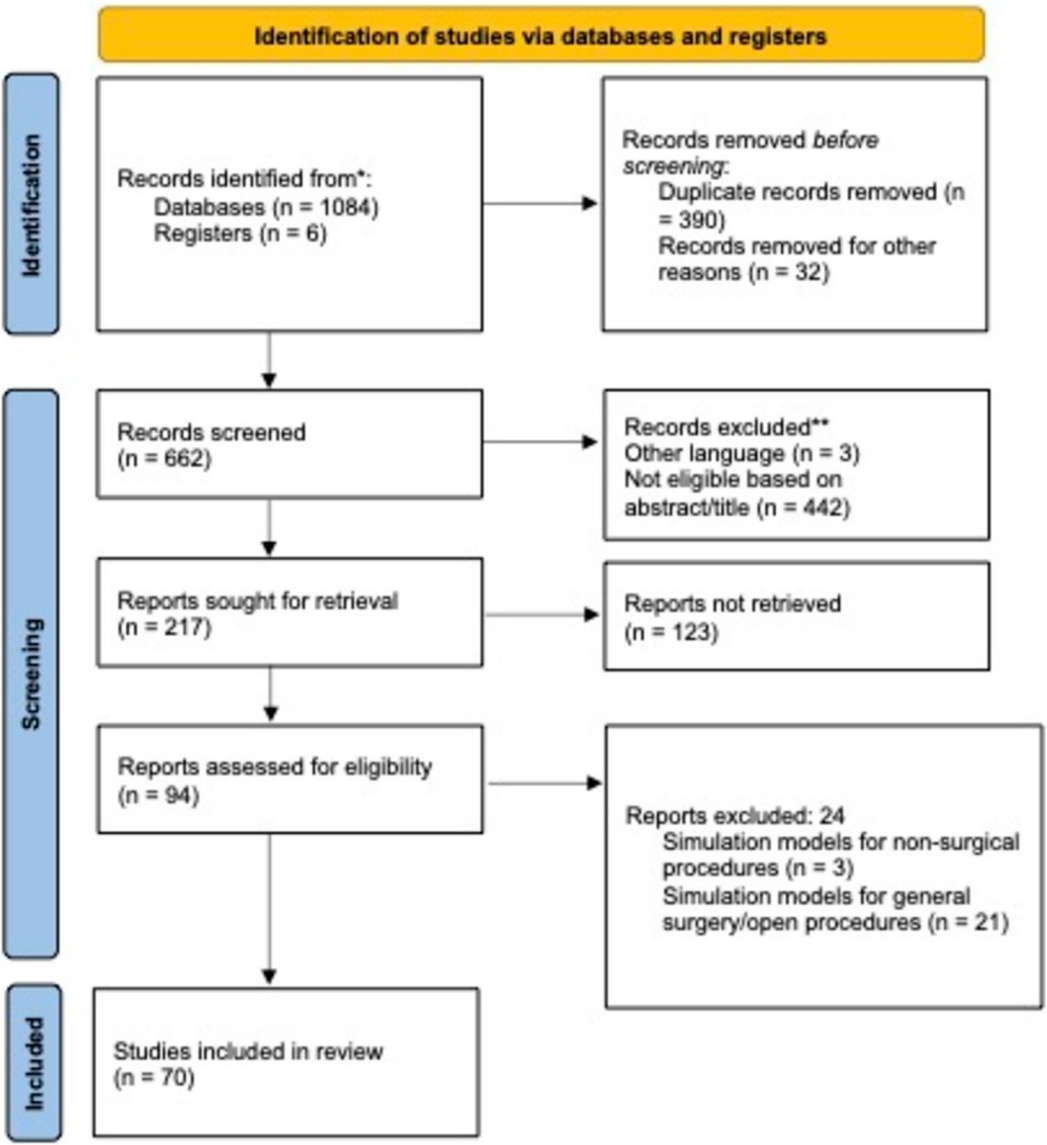
PRISMA flow diagram

The initial search yielded 1084 results. After removing duplicates, 694 titles and abstracts were screened, resulting in the exclusion of 662 studies. Of the 94 full-text articles assessed for eligibility, 72 met the inclusion criteria and were included in the final data extraction (see PRISMA flow diagram in Fig. 1). Full texts of all potentially eligible studies were successfully retrieved using institutional access. Exclusion of the remaining studies was based exclusively on predefined PICOS criteria and not due to access limitations. Results were then categorized into their respective specialty within neonatal/infant surgery: general, gastrointestinal, thorax surgery and urology. All studies included in the review are presented in Table 1.

**Table 1:** Studies included in review

Author/year	Model	Type of model	Participants/experts	Validation	LoE/LoR
Azzie/2011 [14]	PLS simulator FLS simulator	Inanimate MIS box	84/45	PLS scores	2a/B
Bailez/2019 [28]	MIS education program	Low-cost neonatal training training models	842 individually coached workstations	Surgical efficiency Complexity of the procedures	3b/B-C
Ballouhey/2018 [53]	Pyloromyotomy	Simple simulation tool	80/15	Face Content	2a/B-C
Barsness/2013 [42]	CDH	Synthetic	40/6	Face Content Construct	3b/C
Barsness/2014 [47]	EA/TEF	High fidelity synthetic/bovine tissue	40/9	Content Construct	2b/B-C
Barsness/2015 [45]	EA/TEF	High fidelity synthetic (3D printed and silicone)	47/14	Content Construct	2b/C
Barsness/2015 [58]	Duodenal atresia	Inanimate casing with animal tissue (bovine tissue)	18/6	Face Content	2a/C
Barsness/2015 [72]	Lobectomy	3D printed high fidelity synthetic model/animal tissue	33/11	Face Content Construct	2a-b/C
Baumann/2018 [16]	MIS DA, TL, EA/TEF	4-h hands-on course for neonatal MIS	17/1	Pre- and post-training perceived “comfort levels” of MIS skills	2b/B
Bökkerink/2021 [37]	CDH/EA	Low cost, reproducible model, synthetic	60/18	Face Content Construct	2c/B-C
Burdall/2016 [68]	3D choledochal surgery simulator	3D printed synthetic	10/3	Face Content	3/C
Cheung/2014 [75]	Laparoscopic pyeloplasty	3D printed silicon model	27/3	Development Face	2a/B-C
Choi/2022 [51]	EA/TEF	High fidelity 3D printed	17/7	OSATS	2b/B-C
Davis/2013 [87]	EA/TEF	3D neonatal ribcage with fetal/bovine tissue	11/2	Face Content	3/C
Davis/2014 [43]	CDH	Synthetic high-fidelity model	NA	Face Content Construct	3b/C
Deie/2022 [46]	EA	Inanimate/dry box	40/6	Face Content	3/C
Esposito/2016 [26]	Inguinal hernia repair, Varicocelectomy Nephrectomy Fundoplication	Rabbit model versus the porcine	10/0	Operative time Intraoperative complications Surgical performance	3/C
Etlinger/2022 [59]	Duodenal atresia	Animal model (rabbit)	8/7	OSATS	2a/B-C
Fahy/2018 [17]	Intracorporeal suturing task in small and large simulator	Small versus large simulator	100/	Time to task completion Task completion Comparator	4/B
Falcioni/2023 [32]	Laparoscopic ovary sparing surgery (LOSS)	Low-cost model for laparoscopic ovary sparing surgery (LOSS)	83/67	Correlation in time assessment Levels of experience	3b/C
Falcioni/2022 [35]	Telesimulation—Essential Skills Training Module	Telesimulation for MIS	63/30	Explore the effectiveness of T-ESTM	3/B-C
Feng/2015 [88]	Two-dimensional and 3D imaging	Small training boxes using 3D imaging	24/5	Time to task completion Task completion Comparator	2b/B
Gause/2016 [18]	MIS CDH, DA, TL, EA/TEF	Synthetic, 3D printed models + fetal bovine tissue	60/NA	Pre- and post-training perceived "comfort levels" of MIS skills	2a/B
Haehl/2024 [23]	6 MIS training modules	6 MIS training modules	77/61	Construct Content	2b/C
Harada/2015 [71]	Motion sensors for thoracoscopy	Rapid prototyped chest model	30/10	OSATS	2a/B
Hawkinson/2014 [86]	DA/TEF	Low-cost tissue replica	NA	Face	3/C
Heinrich/2006 [84]	Endoscopic small bowel biopsy	In vitro pelvic trainer In vivo animal model	12/0	Comparative	2ba/B-C
Ieiri/2013 [61]	Fundoplication	Inanimate suture ligature model of the crura of the diaphragm for infant fundoplication	16/10	OSATS	2b/B-C
Jhala/2021 [76]	Laparoscopic pyeloplasty	High fidelity animal tissue model	NA	NA	2a/B
Jimbo/2015 [102]	Nissen construction tasks	Box/live tissue	7/0	OSATS	2a/C
Jimbo/2016 [66]	Laparoscopic hepaticojejunostomy simulator	Inanimate MIS box	40/9	Face Content	2a/B-C
Jimbo/2017 [63]	Fundoplication	Laparoscopic simulator	49/15	Face Content Construct	2b/B
Kirlum/2005 [27]	Infantile laparoscopic surgery procedures	Pelvis trainer Rabbit model	12/0	Operating time Suture time and perforation of the bowel	2b/B-C
Kirlum/2005 [74]	Abdominal and thoracic endo-surgery	Animal (rabbit)	NA	Task completion Time suturing	2a/B
Krauss/2009 [65]	Fundoplication	Animal model (pig)	12/0	GOALS	2b/B-C
Krois/2022 [80]	Cysto-vaginoscopy	3D printed cloaca model	41/30	Quality and feasibility of model	2a/B-C
Liddy/2023 [50]	EA/TEF	3D printed synthetic model	17/10	Face Content OSATS	2b/B
Ljuhar/2018 [41]	IH/CDH	Synthetic	107	Face Content Construct	2b/B-C
Maciá/2022 [77]	Laparoscopic pyeloplasty	3D-printed liquid latex model	8/1	OSATS	2b/B
Marecos/2006 [73]	Pleural empyema	Animal (rabbit)	30/30	Face Content	2b/B
Maricic/2016 [31]	EA/TEF	Inanimate low-cost model	39/10	Face Content Construct	2b/B-C
Murakami/2024 [20]	Forceps manipulation Motion analysis	Adult- and infant-sized dry boxes (DBs)	72/0	Task completion Comparator	3/B-C
Nair/2021 [85]	Chest model	Synthetic	25/5	Content	2b/C
Narayanan/2014 [90]	Endoscopic surgery	Porcine non-survival model for endoscopic surgery	114/0	5-point Likert scale	2b/B-C
Nasr/2013 [8]	PLS simulator	Inanimate MIS box	84/45	Construct PLS scores	2b/B
Nasr/2014 [8]	PLS simulator Motion tracking	Inanimate MIS box	75/37	Suture evaluation analysis	2a/B
Obata/2015 [44]	CDH	Synthetic mimicking a newborn's size	29/10	OSATS	2b/B-C
Okata/2024 [92]	Hepaticojejunostomy simulator	Porcine models	8/0	Surgery Skill Assessment System	2b/C
Onishi/2019 [64]	Fundoplication	Dry-box/VR	17/0	GOALS	2a/B
Oquendo/2018 [21]	PLS simulator Motion tracking	Inanimate MIS box	32/0	OSATS	3/B
Ordorica-Flores/2019 [57]	Duodenal atresia	Animal (rabbit)	13/13	Face Content	2a/C
Perez-Merino/2014 [39]	CDH	Animal (rabbits)	20/NA	OSATS	2b/B-C
Plymale/2010 [54]	Pyloromyotomy	Middle fidelity	29/23	Face Content Construct	2b/B-C
PSTRN/2024 [38]	CDH model for thoracotomy and thoracoscopy	3D printed synthetic model	52/20	Measure the diaphragmatic defect and ipsilateral diaphragm	2a/B
Reino-Pires/2018 [36]	CDH	Low cost, do-it-yourself model, synthetic	19/4	Face Content	2c/B-C
Restrepo/2023 [79]	Laparoscopic pyeloplasty	3D printed silicon model	24/NA	Realism, performance Operative time Complications	3/C
Retrosi/2015 [15]	eoSim® simulator Motion analysis	Inanimate MIS box	28/8	Construct Content Concurrent	2a/B-C
Ruiz et al. 2020 [33]	Laparoscopic pyeloplasty, extravesical ureteral reimplantation, Mitrofanoff apendicovesicostomy, and ureteroureteral anastomosis	Pediatric urology low cost models	NA	Facilitate pediatric urology low cost models	2b/B-C
Santos/2012 [69]	Fundamentals of laparoscopic surgery box trainer common bile duct exploration	Inanimate MIS box	21/5	Construct	2b/C
Schwab/2016 [70]	Laparoscopic common bile duct simulator	Inanimate MIS box	30/0	Face Content	3/C
Simforoosh/2011 [91]	Laparoscopic skill training	Dogs Angora rabbits	72/0	Comparative	2b/B
Takazawa/2016 [48]	Rapid prototyped chest model EA/TEF Intracorporeal suturing Knot-tying task	Pediatric chest model	28/9	Task completion Time suturing Errors	2a/B
Takazawa/2016 [48]	Motion sensors for thoracoscopy	Rapid prototyped chest model	53/8	Content Construct	2b/C
Torres /2021 [89]	PLS simulator with sliding tray	Inanimate MIS box	49/14	Face Content	3/B
Trudeau/2017 [83]	PLS simulator Motion tracking Advanced intracorporeal suturing task	Inanimate MIS box	60/39	Suture evaluation analysis	2b/B-C
Weisser/2023 [22]	6 MIS training modules	3D printed SiSiPed 1	25/5	Task completion Technical mistakes	2a/C
Wells/2020 [49]	EA/TEF	High fidelity synthetic (3D printed and silicone)	5/3	Content Construct GOALS	2b/C
Williams/2018 [52]	Pyloromyotomy	High fidelity synthetic (3D printed and silicone)	27/9	Content Construct GOALS TSA	2b/B
Yamada/2022 [62]	Fundoplication	Disease-specific laparoscopic simulator with 3 different display sizes	8/0	Outcomes of endoscopic surgical procedures	2b/B-C
Yamada/2023 [67]	Laparoscopic hepaticojejunostomy simulator	High-fidelity simulator	4/4	OSATS	2b/3
Zahradníková/2023 [4]	EA/TEF	3D printed synthetic model	18/7	Face Content OSATS	2a/B
Zimmerman/2019 [25]	Neonatal laparoscopic surgery	Chicken model	27/9	Face Content	3/B-C

CDH: Congenital diaphragmatic hernia, DA: Duodenal atresia, EA/TEF: Esophageal atresia/tracheoesophageal fistula, TL: Thoracoscopic lobectomy, IH: Inquinal hernia

## Neonatal/infant surgical simulation models

The literature review identified 20 studies, describing 9 models (Table 2). The Pediatric Laparoscopic Surgery Simulators (PLS) were by far the most commonly used, appearing in 7 studies, while 3D-printed synthetic models were used in 5 studies. Preliminary data from Nasr et al. [8], Azzie et al. [14] and Retrosi et al. [15] suggest that PLS simulator remains the only validated tool for pediatric MIS, with construct validity established for its pattern-cutting task through modified scoring metrics that improved differentiation among skill levels. Short exposure courses in neonatal MIS were found to boost surgeons' confidence and perceived skill levels, but they lack the structured deliberate practice necessary for expert performance, emphasizing the need for a mastery learning framework [16, 17]. Validation of the eoSim(®) simulator demonstrated strong content, construct, and concurrent validity, supporting its role in pediatric laparoscopy training, with future efforts needed to implement proficiency-based curricula using validated performance metrics. For all tasks, there were significant differences between level of experience groups for eoSim task completion times and PLS scores [15]. These studies demonstrated (LoE 2b–2a-2b and moderate LoR B-B-B/C, respectively). Gause et. al. [18] showed that Simulation-Based Education (SBE) in pediatric trainees results in significant improvement in both cognitive knowledge and trainee comfort with safe operative techniques for advanced MIS. Training platforms such as the Fundamentals of Laparoscopic Surgery (FLS) have become an integral part of postgraduate adult general surgical education [19]. So far, however, there is no such universal tool for MIS in pediatric patients [20, 21]. Therefore Weisser et al. [22] developed a validated novel 3D printable pediatric MIS simulation program. Thirty-one participants (13 experts and 18 residents) were enrolled in the study (median age: 32 years; 77% male). The expert group outperformed the resident group on all tasks, with all expert surgeons completing them on time, while 33% of residents failed to complete at least one. Laparoscopic suturing experience correlated positively with task scores (P < 0.01). The tasks demonstrated high interrater reliability (0.99) and internal consistency (0.80), with most participants finding them relevant and beneficial for skill development. This study supports the validity of these tasks in assessing suturing skills and suggests their integration into training programs to better prepare residents for surgery [22]. Both studies were rated with the score LoE 2a and LoR B, after establishing a basic 3D-printable pediatric MIS curriculum (SuSiPed1.0) [22]. Haehl et al. developed an advanced pediatric MIS curriculum (SuSiPed2.0) consisting of six training modules. All participants repeated the curriculum three times and evaluated the curriculum regarding closeness to reality and relevance (Likert Scales 0/worst–6/best). This study shows that prior basic training significantly increases the performance in a more advanced pediatric MIS simulator. The authors suggested that both basic and advanced SuSiPed modules can be combined into a comprehensive pediatric MIS training program. It may also be a means of evaluating trainees at different stages of their training [23], both of which attained a level of recommendation of C and level of evidence 2b and 2a retrospectively.

**Table 2:** Neonatal, infant surgical simulation models

Author/year	Model	Type of model	Participants/experts	Validation	LoE/LoR
Azzie/2011 [14]	PLS, FLS simulators	Inanimate MIS box	84/45	PLS scores	2a/B
Baumann/2018 [16]	MIS DA, TL, EA/TEF	4-h hands-on course for neonatal MIS	17/1	Pre- and post-training perceived "comfort levels" of MIS skills	2b/B
Esposito/2016 [26]	Inguinal hernia repair, Varicocelectomy Nephrectomy	Rabbit model versus the porcine	10/0	Operative time Intraoperative complications Surgical performance	3b/C
Fahy/2018 [17]	Intracorporeal suturing task in small and large simulator	Small versus large simulators	105/31	Time to task completion Force analysis parameters Comparator	4/B
Feng/2015 [88]	Two-dimensional and 3D imaging	Small training boxes using 3D imaging	24/5	Time to task completion Force analysis parameters Comparator	2b/B
Gause/2016 [18]	MIS CDH, DA, TL, EA/TEF	Advanced MIS hands-on courses for neonatal MIS	60/NA	Pre- and post-training perceived “comfort levels” of MIS skills	2a/B
Haehl/2024 [23]	6 MIS training modules	Sisi Ped 3D printed synthetic	77/61	Construct Content	2b/C
Hawkinson/2014 [86]	Model evaluation	3D printed synthetic EA + DA	NA	Model evaluation	2b/B-C
Heinrich/2006 [84]	Endoscopic small bowel biopsy	In vitro pelvic trainer In vivo animal model	12/0	Comparative	2b/B-C
Kirlum/2005 [27]	Infantile laparoscopic surgery procedures	Pelvis trainer Rabbit model	12/0	Operating time Suture time and perforation of the bowel	2b/B-C
Murakami/2024 [20]	Forceps manipulation Task: peg transfer	Adult- and infant-sized dry boxes (DBs)	72/0	Task completion time were compared before and after training Comparator	3/B-C
Narayanan/2014 [90]	Endoscopic surgery	Porcine non-survival model for endoscopic surgery	114/0	5-point Likert scale	2b/B-C
Nasr/2013 [8]	PLS simulator Motion tracking	Inanimate MIS box	75/37	Suture evaluation analysis	2a/B
Oquendo/2018 [21]	Pediatric laparoscopic suturing task	Custom pediatric laparoscopic box trainer	32/0	OSATS	3/B-C
Retrosi/2015 [15]	eoSim® simulator Motion analysis	Inanimate MIS box	28/8	Construct Content Concurrent	2b/B-C
Simforoosh/2011 [91]	Laparoscopic skill training	Dogs Angora rabbits	72/0	Comparative	2b/B
Torres /2021 [89]	PLS with sliding tray	Inanimate MIS box	49/14	Face Content	3/B
Trudeau/2017 [83]	PLS simulator Motion tracking Advanced intracorporeal suturing task	Inanimate MIS box	60/39	Suture evaluation analysis	2b/B-C
Weisser/2023 [22]	6 MIS training modules	3D printed SiSi Ped 1	25/5	Task completion Technical mistakes	2a/C
Zimmerman/2019 [25]	Neonatal laparoscopic surgery	Chicken model	27/9	Face Content	3/B-C

CDH: Congenital diaphragmatic hernia, DA: Duodenal atresia, EA/TEF: Esophageal atresia/tracheoesophageal fistula, TL: Thoracoscopic lobectomy

Animal models are used as an alternative method for surgical training due to their ability to mimic human physiology and pathology. Although no model can perfectly replicate the complexity of human biology, animal models provide insights similar to human cadavers in understanding the fundamental mechanisms of diseases [24]. For neonates and infant MIS training, rabbit and avian models are the most frequently utilized. Zimmermann et al. [25] developed a new, cost-effective animal tissue model designed and evaluated for training neonatal MIS skills. A prospective observational study took place during two MAS Skill Labs in June 2018 and April 2019, where fresh chicken cadavers were used for laparoscopic exercises with 3 mm MAS instruments. Twenty-seven participants took part in the study, and their feedback was collected using a 5-point Likert-scale questionnaire based on the Michigan Standard Simulation Experience Scale (MiSSES). Results showed high perceived fidelity, with scores ranging from 3.52 to 4.44 for various laparoscopic aspects, and an overall satisfaction rating of 4.64 ± 0.56. This study successfully validated the avian tissue model as an effective tool for neonatal MAS training, particularly in improving skills such as intracorporeal suturing. They concluded that avian tissue model is very realistic and effective, making it possible to gain laparoscopic skills especially with intracorporeal suturing and knot-tying in a small space [25]. Esposito et al. [26] compared the efficiency of a rabbit versus the porcine model for training in pediatric MIS. Ten young pediatric surgeons underwent training sessions on rabbit and porcine models under the supervision of five experienced tutors. Practitioners were significantly more confident in the rabbit model compared with the pig model, especially for advanced procedures (*P* = 0.03). The overall surgical performance score was significantly higher in the rabbit model compared with the pig model (8.1 versus 6.0; *P* = 0.01). Based on preliminary results authors suggested that, rabbits are preferred over pigs as a training model for pediatric MIS [26]. Kirlum et al. [27] reached a similar conclusion, stating that their experimental study examined how surgical residents performed laparoscopic bowel biopsy and defect repair after training with a pelvis trainer versus a rabbit model. Their findings indicated that repetitive training using a rabbit model led to superior skills for live operations. The authors concluded that in pediatric surgical centers specializing in advanced laparoscopic procedures, incorporating an animal model into training should be considered an essential step as it may contribute to better patient outcomes [27]. Overall, these studies demonstrate moderate evidence (LoE 2b–3b) and moderate recommendations (LoR B-C) for integrating cost-effective and animal-based models into surgical training.

## Low-cost validated models and the experience of using them in a pediatric surgery training program

Simulation-based training is a cornerstone of minimally invasive pediatric surgery (MIPS) education. In environments where resources may be limited, the development and the use of low-cost validated models have proven to be both feasible and impactful [28]. At its foundation is low-cost, handmade simulation models, many of which have been designed and validated internally by teams comprising surgical fellows, scrub nurses, and technicians. Several of these models have been published in peer-reviewed journals and adopted by other institutions, leading to collaborative exchanges and refinement of training methodologies [29].

Bailez et al. developed a structured pediatric and neonatal MIS (PNMIS) simulation program in 2012, incorporating a three-stage curriculum and an MIS education fellowship to build surgical proficiency through supervised, low-cost models, and formative feedback. The program has delivered 184 on-site and 41 telesimulation courses, demonstrating measurable improvements in participants’ surgical efficiency and procedure complexity, with 75% reporting significant gains. Its success has led to academic output, international replication, and growing interest in its scalable, evidence-based approach to simulation-based MIS training [30]. Maricic et al. created and validated a low-cost, inanimate thoracoscopic model for training in the repair of esophageal atresia with tracheoesophageal fistula (EA/TEF), using household materials to replicate neonatal thoracic anatomy. Thirty-nine pediatric surgeons with varying levels of experience tested the model, and the majority rated its anatomical fidelity and educational value highly, particularly for skill acquisition in EA/TEF repair. Performance metrics showed that more-experienced surgeons performed faster procedures with fewer errors and better anastomoses, supporting the model’s utility as a valid training tool now also used in full neonatal MIS team simulations [31]. Falcioni et al. demonstrated that a low-cost simulation model for laparoscopic ovary-sparing surgery (LOSS) in pediatric ovarian teratomas is an effective training tool, with high ratings for anatomical accuracy and skill development. This study found a significant correlation between participants’ prior experience and their performance, supporting the integration of such models into simulation curricula to enhance surgical proficiency and reduce complications [32].

Ruiz et al. developed four low-cost, 3D-printed pediatric urology models—integrated into the “Gruyere” trainer—to simulate key minimally invasive procedures, providing an effective, portable, and structured tool for enhancing surgical skills within fellowship training programs [33]. Falcioni et al. evaluated the effectiveness of a Tele-assisted Essential Skills Training Module (T-ESTM) developed during the COVID-19 pandemic to ensure continuity of minimally invasive surgery (MIS) training. Across two studies, T-ESTM demonstrated significant improvements in participants' technical skills and task efficiency, with results comparable to those achieved through standard on-site simulation. These findings support telesimulation as a reproducible, effective, and scalable alternative to traditional simulation-based surgical education [34, 35].

## Congenital diaphragmatic hernia (CDH) surgery models

The analyzed studies focus on low-cost, animal-based, and box trainer models designed specifically for CDH simulation (Table 3). Several studies have validated low-cost, do-it-yourself (DIY) models for thoracoscopic training. Reino-Pires et al. [36] introduced a neonatal thoracoscopic CDH repair model constructed from household materials, demonstrating high feasibility and positive user feedback, with validation performed through their own assessment system [36]. Similarly, Bökkerink et al. [37] evaluated cost-effective models for CDH and esophageal atresia (EA) training, confirming their suitability for skill acquisition based on structured participant feedback. However, further comparative studies and objective skill validation are needed to assess their long-term educational impact [37], both of which attained a level of recommendation of C/B and level of evidence 2c. Pediatric Surgical Trainees Research Network (PSTRN) [38] aimed to develop a 3D-printed model of CDH and test interobserver variability in the simulated model for obtaining measurements of the diaphragmatic defect and ipsilateral diaphragm. The authors concluded that these 3D-printed models provide a valuable tool for surgical training and standardizing measurements in CDH repair. Their reproducibility across different experience levels supports their potential integration into medical education and skills assessment [38]. Animal models have been extensively explored for CDH training. Pérez-Merino et al. [39] and Takimoto et al. [40] developed standardized rabbit models for thoracoscopic CDH repair, confirming their anatomical accuracy and surgical fidelity. Their study effectively differentiated novices from experts based on task completion time, complication rates, and suture quality. The model received high ratings for realism and surgical applicability. The authors concluded that laparoscopic box trainers provide an accessible alternative for structured skill assessment [39, 40]. Pérez-Merino et al. [39] optimized the hernia development timeframe to 48 h, balancing realistic anatomy with minimal animal stress. Validation by six pediatric surgeons confirmed its surgical fidelity and educational value though comparative validation against other models and long-term impact assessment were not included [39]. Both studies evaluated surgical assessment scoring and were assigned LoE 2b and LoR B/C for both models. Ljuhar et al. [41] introduced the Laparoscopic Inguinal and Diaphragmatic Defect (LIDD) model, validated on 107 participants. This study confirmed construct validity, demonstrating significant skill differentiation between novices, trainees, and experts. However, the model did not distinguish between different levels within the intermediate group and lacked direct comparison with other simulation tools [41]. Several dedicated CDH repair simulators have been proposed. Barsness et al. [42] and Davis et al. [43] developed low-cost thoracoscopic CDH repair models, iterating on 3D-printed neonatal rib cages, synthetic diaphragms, and tissue substitutes. These models received high ratings for relevance and training value, but fidelity—especially in terms of haptic feedback—remained a limitation. Both studies emphasized the importance of iterative model refinement based on user feedback but did not include objective performance validation metrics [42, 43]. Obata et al. [44] took a quantitative validation approach, incorporating instrument tracking, forceps movement analysis, and objective suture quality assessment. Their study differentiated experts from trainees based on task completion time, suturing precision, forceps movement efficiency, and biomechanical tissue response. The model effectively identified disparities in skill levels, particularly in bilateral forceps coordination, suggesting its potential for standardized skill validation in pediatric thoracoscopic training [44]. Overall, these studies demonstrate LoE 2b–3b, and moderate LoR B-C for integrating cost-effective and animal-based models into surgical training. While promising, further comparative research and skill transfer studies are necessary to determine the optimal model for structured pediatric thoracoscopic education.

**Table 3:** Congenital diaphragmatic hernia surgery models

Year/author	Model	Type of model	Participants/experts	Validation	LoE/LoR
Barsness/2013 [42]	CDH	Synthetic	40/6	Face Content Construct	3b/C
Bökkerink/2021 [37]	CDH/EA	Low cost, reproducible model, synthetic	60/18	Face Content Construct	2c/ B-C
Davis/2014 [43]	CDH	Synthetic high fidelity	NA	Face Content Construct	3b/C
Ljuhar/2018 [41]	IH/CDH	Synthetic	107	Face, content, construct	2b/B-C
Obata/2015 [44]	CDH	Synthetic mimicking a newborn's size	29/10	OSATS	2b/B-C
Pérez-Merino/2014 [39]	CDH	Animal (rabbits)	20/NA	OSATS	2b/B-C
PSTRN/2024 [38]	CDH model for thoracotomy and thoracoscopy	3D printed synthetic model	52/20	Measure the diaphragmatic defect and ipsilateral diaphragm	2a/B
Reino-Pires/2018 [36]	CDH	Low cost, do-it-yourself model, synthetic	19/4	Face Content	2c/B-C

## Esophageal atresia/tracheoesophageal fistula (EA/TEF) surgery models

A total of 10 articles about EA/TEF models were identified from the used databases (Table 4).

**Table 4:** Esophageal atresia, tracheoesophageal fistula models

Author/Year	Model	Type of model	Participants/experts	Validation	LoE/LoR
Barsness/2014 [47]	EA/TEF	High fidelity synthetic/bovine tissue	40/9	Content Construct	2b/B-C
Barsness/2015 [45]	EA/TEF	High fidelity synthetic (3D printed and silicone)	47/14	Content Construct	2b/C
Davis/2013 [87]	EA/TEF	3D neonatal ribcage with fetal/bovine tissue	11/2	Face Content	3/C
Deie/2022 [46]	EA	Inanimate/dry box	40/6	Face Content	3/C
Hawkinson/2014 [86]	DA, TEF	Low cost tissue replica	NA	Face	3/C
Choi/2022 [51]	EA/TEF	High fidelity 3D printed	17/7	OSATS	2b/B-C
Liddy/2023 [50]	EA/TEF	3D printed synthetic model	17/10	Face Content OSATS	2b/B
Maricic/2016 [31]	EA/TEF	Inanimate low cost model	39/10	Face Content Construct	2b/B-C
Wells/2011 [49]	EA/TEF	High fidelity synthetic (3D printed and silicone)	5/3	Content Construct GOALS	2b/C
Zahradníková/2023 [4]	EA/TEF	3D printed synthetic model	18/7	Face Content OSATS	2a/B

Forty percent of the studies were recent, with four out of ten published in the last five years (2020–2024). The authors agreed that there were currently no validated simulation tools commercially available to help train pediatric surgery trainees with this technically challenging surgical procedure[45–47]. All studies discussed the development and the application of their own simulators and emphasize that the development of the simulator should be low-cost and the model reusable, avoiding the use of animal tissues [48, 49]. Most model authors involved students and trainees for testing. Three studies, Barsness et al., Choi et al., and Liddy et al., have shown that the EA/TEF model is suitable both for improving the clinical practice of experts in pediatric surgery and as a suitable learning tool for novices [47, 50, 51]. These studies provide moderate-level evidence (LoE 2b–3b) and support moderate-strength recommendations (LoR B-C) for incorporating cost-effective models into surgical training, while also emphasizing the appropriateness of including EA/TEF simulators in neonatal/infant surgical MIS training.

## Upper gastrointestinal (GI) tract surgery models

A total of 12 articles were identified from the used databases (Table 5). Williams et al. [52] created 3D-printed organs to practice surgical techniques. This study validated a 3D-printed hypertrophic pyloric stenosis (HPS) stomach model by assessing its reliability and surgical realism through graded laparoscopic pyloromyotomy (LP) attempts by medical trainees and surgeons. Results showed significant skill improvement, high interrater reliability, and strong participant agreement on the model’s accuracy and usefulness for training [52]. A similar result was reported by Ballouhey et al. [53], who validated their study using the OSATS score and the reproducibility of the model construction. A cohort of experts completed a six-item questionnaire on a four-point scale, evaluating the model's realism and accuracy. HPS model was created and integrated into a pediatric laparoscopic surgery simulator, demonstrating sufficient accuracy for teaching LP. Based on these findings, it can be considered an effective tool for LP simulation training in the educational program for fellows [53]. Overall, these studies demonstrate moderate evidence (LoE 2a–2b) and moderate recommendations (LoR B-C) for integrating cost-effective and animal-based models into surgical training. Plymale et al. demonstrated that their inanimate LP trainer effectively simulates key procedural steps, as validated by pediatric surgeons. The model provided a valuable training experience for medical students and residents, with significant improvements in knowledge and skills immediately post-training. However, while technical skills were retained after two months, cognitive knowledge retention was less sustained [54, 55].

**Table 5:** Upper gastrointestinal tract surgery models

Author/year	Model	Type of model	Participants/experts	Validation	LoE/LoR
Ballouhey/2018 [53]	Pyloromyotomy	Simple simulation tool	80/15	Face Contect	2a/B-C
Barsness/2015 [58]	Duodenal atresia	Inanimate casing with animal tissue (bovine tissue)	18/6	Face Content	2a/C
Etlinger/2022 [59]	Duodenal atresa	Animal model (rabbit)	8/7	OSATS	2a/B-C
Ieiri/2013 [61]	Fundoplication	Inanimate suture ligature model of the crura of the diaphragm for infant fundoplication	16/10	OSATS	2b/B-C
Jimbo/2015 102]	Nissen construction tasks	Box/live tissue	7/0	OSATS	2a/C
Jimbo/2017 [63]	Fundoplication	Laparoscopic simulator	49/15	Face Content Construct	2b/B
Krauss/2009 [65]	Fundoplication	Animal model (pig)	12	GOALS	2b/B-C
Onishi/2019 [64]	Fundoplication	Dry-box/VR	17/0	GOALS	2a/B
Ordorica-Flores/2019 [57]	Duodenal atresia	Animal (rabbit)	13/13	Face Content	2a/C
Plymale/2010 [54]	Pyloromyotomy	Middle fidelity	29/23	Face Content Construct	2b/B-C
Williams/2018 [52]	Pyloromyotomy	High fidelity synthetic (3D printed and silicone)	27/9	Content Construct GOALS TSA	2b/B
Yamada/2022 [62]	Fundoplication	Disease-specific laparoscopic simulator with 3 different display sizes	8/0	Outcomes of endoscopic surgical procedures	2b/B-C

**Training for laparoscopic duodenal atresia (DA)** is difficult, extending operative times and increasing costs. Traditional training relies on apprenticeship, which is less effective due to limited cases. Simulation is essential, allowing repeated practice and error correction in a controlled environment. High-fidelity simulators are needed to replicate neonatal conditions and develop technical competence, especially for neonatal MIS [56]. Ordorica-Flores et al. [57] describe the validation process of a rabbit model (weighing 3.0–4.5 kg) with face validity (96%) and content validity (84%) [57]. Barsness et al. [58] describe a model consisting of an inanimate casing with fetal bovine tissue for the organs. They describe face validity (88%) and content validity (95%) [58]. Etlinger et al. [59] described a rabbit model of laparoscopic duodenum atresia surgery involving a diamond-shaped duodeno-duodenostomy, while their data confirm the feasibility of this advanced pediatric laparoscopic model [59]. All three studies confirmed the feasibility and educational value of animal or hybrid models for laparoscopic duodenal atresia repair. Both studies evaluated surgical assessment scoring and were assigned LoE 2a for both models respectively, and LoR C-B/C respectively. Rabbit-based and tissue-based simulations demonstrated high realism and utility for training. Further validation and standardization are needed to support broader adoption in pediatric surgical education.

**Laparoscopic fundoplication (LF)** has almost uniformly replaced open fundoplication (OF) in children. Shorter hospital stays, less postoperative pain, and fewer complications are highlighted as the main advantages of LF as compared to OF [60]. However, pediatric surgeons require highly advanced skills when performing endoscopic surgery; hence, their experience is often limited in comparison to general surgeons [61, 62]. Jimbo et al. aimed to evaluate the effectiveness of endoscopic surgery training for less-experienced pediatric surgeons and then compare their skills before and after training [63]. Seven young pediatric surgeons completed a 2-day endoscopic skill training program, including lectures, box training, and live tissue training, and their performance on Nissen construction tasks was objectively evaluated before and after training using statistical analysis. The short-term training program effectively improved pediatric surgery trainees' endoscopic skills, as demonstrated by enhanced task performance and better instrument control [63]. Jimbo et al. [43] validated a similarly designed simulator. A dedicated laparoscopic fundoplication simulator was used to evaluate and compare the technical performance of general surgeons (GS) and pediatric surgeons (PSE). Both groups demonstrated excellent bimanual coordination and were able to manipulate both forceps effectively. However, PSE surgeons performed the most compact and precise maneuvers within the confined operative space, indicating a higher level of spatial efficiency in challenging conditions. Onishi et al. [64] compared three endoscopic surgical training programs for medical students using an infant laparoscopic fundoplication simulator. They developed a 10 kg infant body model based on CT data and established a pneumoperitoneum model to simulate clinical conditions. Results showed that the virtual group's assistant forceps had a longer path length and faster acceleration, suggesting the need for long-term, combined training for medical students and young surgeons [64]. The model developed by Jimbo et al. was rated as LoE 2a and LoR C. Similarly, the infant laparoscopic fundoplication simulator created by Onishin et al. was evaluated as LoE 2a and LoR B. Krauss et al. [65] developed a computer-based surgical workflow analysis module for LF and evaluated its applicability in an infant pig model. Using synchronized intra- and extracorporeal recordings, segmented the procedure into four phases and recorded specific tasks on a virtual timeline to assess time variations. Their findings showed significant reductions in preparation and dissection times, while reconstruction and conclusion phases, as well as knot-tying, remained stable, demonstrating the potential of this model for refining surgical techniques and training evaluation [65].

## Hepatobiliary surgery models

Six studies refer to this field, three of which described hepatobiliary laparoscopic surgery training on inanimate MIS box (Table 6). There were also two studies that aim to evaluate the 3D printed models [66, 67]. Burdall et al. [68] developed 3D printed hybrid model, harnessing 3D technology to simulate laparoscopic choledochal surgery [68]. Digital hepatic anatomy images and standard laparoscopic trainer dimensions were employed to create an entry level laparoscopic choledochal surgery model. Ten senior pediatric surgical trainees participated in this study, The session consisted of a 20 min simulated excision focusing on four key steps: (I) active traction and then division of the common bile duct; (ii) dissection of the balloon cyst from the sponge bed; (iii) internal visualization of the upper limit of the cyst and the duct level and (iv) transaction of the cyst and hybrid anastomosis with porcine esophagus as the simulated roux loop. Feedback was then collected from delegates on three key areas using visual analog scores: tactile feedback, usefulness and complexity. The simulation received mixed feedback on fidelity, scoring an average of 5.6/10 (moderate similarity to the real operation). The complexity was deemed appropriate, with a mean score of 6.2/10. Overall, it was considered highly useful, scoring 7.36/10. Additionally, all delegates agreed it was easily reproducible, and 100% would recommend it to their colleagues [68]. Overall, this study demonstrated face and content validity and was given an LoE of 3 and LoR C. While further refinement is needed, 3D printing shows great potential for developing detailed simulations of rare and complex surgeries [68]. Santos et al. [69] created a procedure algorithm and low-cost laparoscopic common bile duct exploration (LCBDE) simulator incorporating the laparoscopic, endoscopic, and fluoroscopic views [69]. Subsequently, Schwab et al. [70] sought to evaluate a previously developed general surgery LCBDE simulator developed by Santos et al. among a cohort of pediatric surgical trainees. 30 participants performed a transcystic LCBDE using a simulator and evaluated it with a 28-item instrument assessing Quality and Ability to Perform. Results showed high ratings for operative value (OA = 3.79) but lower ratings for physical/visual attributes (OA = 3.19), with favorable Rasch indices and interitem consistency (*α* = 0.94 for quality, 0.56 for ability). Participants found the simulator valuable for pediatric LCBDE training, suggesting it could be used with minor improvements [70]. Both models received a level of recommendation (LoR) of C and level of evidence 2b and 3, respectively.

**Table 6:** Hepatobiliary tract surgery models

Author/year	Model	Type of model	Participants/experts	Validation	LoE/LoR
Burdall/2016 [68]	3D printed	3D choledocal surgery simulator	10/3	Face Content	3/4
Jimbo/2016 [66]	Inanimate MIS box	Laparoscopic hepaticojejunostomy simulator	40/9	Face Content	2a/3
Okata/2024 [92]	Porcine models	Hepaticojejunostomy simulator	8/0	A-Lap Mini Endoscopic Surgery Skill Assessment System	2b/3
Santos/2012 [69]	Inanimate MIS box	Fundamentals of laparoscopic surgery box trainer common bile duct exploration	21/5	Construct	2b/3
Schwab/2016 [70]	Inanimate MIS box	Laparoscopic common bile duct simulator	30/0	Face Content	3/3
Yamada/2023 [67]	High-fidelity simulator	Laparoscopic hepaticojejunostomy simulator	4/4	OSATS	2b/3

## Thoracic surgery models

Seven articles describing models for thoracic surgery were identified, whereas four validation studies were identified (Table 7). These included rapid prototyped chest model described by Takazawa et al. [48] and Harada et al. [71] developed and demonstrated content and construct validity for a Rapid prototyped chest model. A total of 53 surgeons participated in Takazawa et al. study. Each performed an endoscopic intracorporeal suturing and knot-tying task on both a 3D-printed pediatric chest model with embedded force sensors and a conventional box trainer. Skilled surgeons outperformed unskilled ones in six key metrics using the pediatric simulator, while no significant differences were found in the box trainer. The simulator effectively distinguished skill levels and offers valuable benchmarks for pediatric surgical training [48]. In the study by Barsness et al. [72], fetal bovine tissues were used to complement the 3D-printed high-fidelity synthetic model simulator. Thirty-three participants performed the simulated thoracoscopic lobectomy. Participants completed a 26-item self-report instrument, with validity evidence evaluated using the many-facet Rasch model and internal consistency measured by Cronbach's alpha (*α* = 0.90). Experienced surgeons rated the simulator slightly higher than novices (3.6 vs. 3.4, *P* = 0.001), with high ratings for Physical Attributes (3.7) and a global rating (2.9), suggesting it was useful but could be improved. Barsness et al. lobectomy simulator demonstrated both face, content and construct validity and was given an LoE 3a-b LoR of C [72]. Two articles described animal models which simulate chest pathology for thoracic endo-surgery. The first, developed by Marecos et al. [73] demonstrated usefulness of an experimental empyema in rabbits as a thoracoscopic training model. 20 New Zealand rabbits were anesthetized, and empyema was induced by injecting turpentine, saline, *E*. *coli*, and agar before surgical debridement by 30 pediatric surgeons in a laparoscopic course. 17 rabbits (85%) developed fibrinopurulent empyema, and 76.7% of surgeons rated the model as very good, confirming its usefulness for thoracoscopic training [73]. The second model described by Kirlum et al. used New Zealand white rabbits, with anatomy comparable to neonates, allowed surgeons to practice key techniques, such as gastrostomy, colostomy, and lung biopsies. This model helped refine technical skills and could enhance surgical training in pediatric endo-surgery [74]. The model developed by Marecos et al. was rated as LoE 2b and LoR B. Similarly, the abdominal and thoracic endo-surgery simulator created by Kirlum et al. was evaluated as LoE 2a and LoR B.

**Table 7:** Thoracis surgery models

Author/year	Model	Type of model	Participants/experts	Validation	LoE/LoR
Barsness/2015 [72]	Lobectomy	3D printed high fidelity synthetic model/animal tissue	33/11	Content Construct	2a-b/C
Harada/2015 [71]	Motion sensors for thoracoscopy	Rapid prototyped chest model	30/10	OSATS	2a/B
Kirlum/2005 [74]	Abdominal and thoracic endosurgery	Animal (rabbit)	NA	Task completion Time suturing	2a/B
Marecos/2006 [73]	Pleural empyema	Animal (rabbit)	30/30	Face Content	2b/B
Nair/2021 [85]	Synthetic	Chest model	25/5	Content	2b/C
Takazawa/2015 [48]	Rapid prototyped chest model/EA/TEF intracorporeal suturing and knot-tying task	Pediatric chest model	28/9	Task completion Time suturing Errors	2a/B
Takazawa/2016 [48]	Motion sensors for thoracoscopy	Rapid prototyped chest model	53/8	Content Construct	2b/C

## Urological surgery models

Overall, five validation studies describing models usable for MIS training in urology were identical. Four synthetic models and one using animal tissue (Table 8). The authors who developed 3D-printed synthetic models for laparoscopic pyeloplasty suggested that high-fidelity, low-cost, and reusable models are effective for training surgeons in pediatric pyeloplasty. Across different materials—silicone, liquid latex, and dry-laboratory 3D-printed models, these simulators demonstrated strong realism, usability, and educational value, with promising validation results from both trainees and experienced surgeons [75–77]. These findings support the integration of 3D-printed and synthetic models into laparoscopic training programs to improve surgical skills and enhance patient outcomes in pediatric urology [75, 78, 79]. Krois et al. [80] aimed to evaluate the quality and feasibility of a real-size 3D-printed cloaca model for the purpose of cysto-vaginoscopy evaluation. Forty-one participants rated the patient-specific 3D-printed cloaca model as high-quality and feasible for preoperative training, with 85.4% recommending its use [80]. Patient-specific 3D-printed models demonstrate high feasibility and quality for preoperative evaluation and training in complex anorectal malformations, providing an excellent anatomical view for cysto-vaginoscopy simulation. These models offer a valuable addition to specialty training in pediatric colorectal surgery, enhancing anatomical understanding and surgical confidence. Their effectiveness was evaluated with level of evidence 2a and level of recommendation B–C.

**Table 8:** Urological surgery models

Year/author	Model	Type of model	Participants/experts	Validation	LoE/LoR
Cheung/2014 [75]	Laparoscopic pyeloplasty	3D printed silicon model	27/3	Development Face	2a/B-C
Jhala/2021 [76]	Laparoscopic pyeloplasty	High fidelity animal tissue model	NA	NA	2a/B
Krois/2022 [80]	Cysto-vaginoscopy	3D printed cloaca model	41/30	Quality and feasibility of a real-size 3D-printed cloaca model	2a/B
Maciá/2022 [77]	Laparoscopic pyeloplasty	3D-printed liquid latex model	8/NA	OSATS	2b/B
Restrepo/2023 [79]	Laparoscopic pyeloplasty	3D printed silicon model	24/NA	Realism Performance Operative time Complications	3/C

## Discussion

Simulation-based training has emerged as a valuable tool in enhancing surgical safety, particularly in pediatrics MIS [81]. Despite its growing importance, there are currently no internationally standardized practical examination frameworks for pediatrics surgery. While simulation is now an established component of general surgical training, pediatrics surgery and especially neonatal MIS still lag behind. Performing MIS in neonates and infants poses unique challenges given the limited anatomical space and the fragility of neonatal tissues [82]. These technical demands highlight the necessity for structured and validated training pathways, which remain undefined for this population [83–85]. This systematic review aimed to identify currently available training models for neonatal and infant MIS and evaluate their validity based on the published literature.

Using PRISMA guidelines, 70 studies were included, each describing different models developed for MIS training. The majority of the identified procedures were endoscopic or laparoscopic in nature, and most enrolled subjects were surgical residents (Table 1). Nasr et al. [8], Azzie et al. [14], and Retrosi et al. [15] found that PLS were the most frequently used models, with strong construct and content validity established for their use in skill differentiation. However, short-exposure courses were insufficient to establish mastery learning, emphasizing the need for structured, proficiency-based curricula. Similarly, Weisser et al. [22] demonstrated that SBE in pediatrics trainees results in significant improvement in both cognitive knowledge and trainee comfort with safe operative techniques for advanced MIS. Gause et al. [18] highlighted the effectiveness of SBT in improving both technical skills and trainee confidence, further reinforcing the importance of repeated deliberate practice. Many authors have integrated 3D printing to demonstrate the advantages of three-dimensional (3D) vision in laparoscopic training, particularly in small working spaces, which are common in pediatrics surgery [4, 22, 23, 47, 49, 86, 87]. Feng et al. [88] highlighted that surgical novices and residents benefit significantly from 3D vision as their findings indicate improved performance times and reduced errors compared to two-dimensional imaging. While experts did not exhibit significant differences in error rates or completion times, their suturing efficiency improved in constrained environments, suggesting a role for 3D imaging in refining complex motor skills [88]. These findings align with Hawkinson et al. [86] and Weisser et al. [22], who emphasized the value of 3D-printed, cost-effective, and anatomically realistic models in pediatrics surgical education. The integration of 3D printing with 3D vision simulation could further enhance the fidelity and the accessibility of training by providing customizable, portable, and standardized tools for MIS training. This aligns with the work of Haehl et al. [23], who showed that structured 3D-printable training curricula improve both basic and advanced pediatrics MIS performance. Therefore, combining 3D vision technology with 3D-printed models could be a transformative approach, fostering enhanced skill acquisition while addressing cost and accessibility challenges in surgical education [89].

## Role of animal models in pediatrics MIS training

Animal models continue to play a significant role in pediatrics MIS training due to their ability to replicate human anatomical structures and surgical conditions. Multiple studies have demonstrated their effectiveness in skill acquisition, particularly for procedures where synthetic or inanimate models lack the required physiological accuracy [25, 57, 59, 65, 90–92]. However, their long-term applicability remains debated due to ethical concerns, cost constraints, and the emergence of advanced synthetic models. Esposito et al. [26] and Kirlum et al. [74] validated rabbit models for pediatrics laparoscopic procedures, confirming their ability to replicate neonatal anatomy effectively. Esposito et al. compared rabbit and porcine models for pediatrics MIS training and found that participants demonstrated significantly higher confidence and skill acquisition using the rabbit model (*P* = 0.03). The overall surgical performance score was also superior in the rabbit model compared to the pig model (8.1 vs. 6.0; *P* = 0.01), supporting its preference for advanced training [26]. Similarly, Kirlum et al. [27] concluded that repetitive training on rabbit models led to superior laparoscopic bowel biopsy and defect repair skills compared to traditional pelvic trainers [27]. Pérez-Merino et al. [36] and Takimoto et al. [40] further explored rabbit models for CDH training. Their studies confirmed that these models accurately replicate thoracoscopic conditions, allowing pediatrics surgeons to develop essential technical skills. Pérez-Merino et al. [39] optimized the hernia development timeframe to 48 h, ensuring realistic anatomy with minimal animal stress, while Usoón-Casaús et al. validated the model’s ability to distinguish between novice and expert performance based on task completion time and suture quality [36, 40]. For thoracoscopic training, Marecos et al. [73] developed an experimental pleural empyema model using New Zealand rabbits, assessing its utility among 30 pediatrics surgeons. The majority (76.7%) rated the model as very good, highlighting its effectiveness in enhancing endoscopic debridement techniques [73]. Similarly, Kirlum et al. [74] used rabbit models to refine gastrostomy, colostomy, and lung biopsy techniques, concluding that these models offer a valuable bridge between basic training and live surgery. Zimmermann et al. [25] introduced an avian tissue model for neonatal laparoscopic training, particularly focusing on intracorporeal suturing. Their prospective observational study, conducted with 27 participants, demonstrated high perceived realism (Likert scores 3.52–4.44) and overall satisfaction (4.64 ± 0.56), reinforcing its potential as a cost-effective alternative to live animal models [25]. In contrast, Narayanan et al. [90] validated a porcine-based endoscopic training model, demonstrating its effectiveness in skill acquisition but highlighting concerns about cost and accessibility. Comparing validation methods, most animal models were assessed using OSATS and Likert-scale participant feedback. Rabbit models consistently scored high for realism and skill differentiation, particularly in complex neonatal procedures [90]. However, synthetic and 3D-printed models, such as those developed by Barsness et al. and Choi et al., offer reusable, cost-effective alternatives with promising validation metrics. While animal models provide superior physiological accuracy, the trend in pediatrics surgical education is shifting toward ethically sustainable and technologically advanced solutions [51, 58].

Despite advances in 3D printing and silicone molding, there remain substantial shortcomings in replicating the complexity, realism, and variability inherent in natural tissues and disease processes. Therefore, animal models—particularly in advanced training and mastery-level courses—will remain the gold standard until 3D-printed models can overcome these limitations [93]. Across the world, governmental bodies and institutional review boards have developed legislation and ethical approval processes to limit current animal-based research studies to those where all alternatives are fully considered and animal suffering is minimized [94]. As research involving animals progresses, it is essential to prioritize and uphold the principles of the "three R's" of animal research: reduction, refinement, and replacement [95]. The use of animals should be kept to a minimum whenever possible, with alternatives employed whenever they are available. Notably, countries like the Netherlands are already taking steps toward phasing out animal testing for medications and chemicals [96].

## Contribution and limitations of VR simulators in the development of a training program

However, the virtual reality (VR) in surgical training should be also mentioned. VR technologies are becoming more accessible and are a potential cognitive enhancer in the field of surgical education [97]. The use of VR has been favored in the surgical education, due to many reasons, such as lack of mentors, reduction in training hours and various issues concerning operative procedures [98]. VR provides a safe, interactive learning environment that provides a necessary adjuvant to more traditional passive lecture-based learning, which remains common in surgical training programs. Active learning methods, including VR simulation, improve acquisition of knowledge, increase motivation for self-directed learning and also increase the transfer of skills to clinical practice. [99–101]. Limitations of the use of VR may appear in the evaluation of the learning curve and in older adults due to lack of acquaintance with the technology [97].

## Training models for specific MIS procedures

The findings demonstrate that low-cost synthetic models have gained attention for CDH training, with studies by Reino-Pires et al. and Bökkerink et al. confirming their feasibility and positive user feedback [36, 37]. Similar to previous studies, our findings indicated that these cost-effective models are suitable for skill acquisition, especially in resource-limited settings. However, a major limitation is the lack of objective validation metrics and comparative studies assessing their skill transferability to clinical practice. In contrast, animal models, such as those developed by Pérez-Merino et al. (2014) and Usón-Casaús et al. (2014), provide a high degree of anatomical realism [39, 40]. Based on our findings, these models effectively differentiate novice and expert performance based on metrics like task completion time and suture quality. Conversely, ethical concerns, cost, and regulatory challenges associated with live animal models limit their widespread adoption. Synthetic box trainers, such as the Laparoscopic Inguinal and Diaphragmatic Defect (LIDD) model introduced by Ljuhar et al. (2018), offer a structured assessment of thoracoscopic skills. The results showed that this model successfully differentiated between novices, trainees, and experts. Thus, it presents a viable alternative to animal models [41]. However, it did not clearly distinguish proficiency within intermediate learners and was not directly compared to other simulation tools, leaving gaps in its validation. Overall, these connections highlight the need for comparative studies evaluating the effectiveness of synthetic, low-cost, and animal-based models to determine their role in structured pediatrics thoracoscopic education.

In addition, recent research highlights the lack of standardized EA/TEF simulation tools for pediatrics surgery training [86]. Studies by Barsness et al. (2014), Choi et al. (2022), and Liddy et al. (2023) demonstrated that their models improved both novice and expert skill sets, reinforcing their relevance in EA/TEF training [47, 50, 51]. Relevant studies favor models that are low-cost and reusable, with a preference for 3D-printed synthetic designs over animal-based tissues. The results showed that motion-tracking technology, as implemented by Choi et al. (2022), offers objective performance assessment by measuring forceps movement and task completion time [51]. Conversely, traditional evaluation methods, such as self-reported feedback and OSATS scoring, still dominate the field, indicating a need for more quantitative validation methods. Thus, further integration of motion-tracking, force analysis, and structured assessment tools could significantly enhance the educational value of EA/TEF simulation models.

For pyloromyotomy training, Williams et al. and Ballouhey et al. validated 3D-printed hypertrophic pyloric stenosis (HPS) models, demonstrating high interrater reliability and skill improvement among trainees [52, 53]. Based on the findings, these models offer an effective and reproducible method for training laparoscopic pyloromyotomy. However, Plymale et al. observed that while technical skills were retained, cognitive knowledge declined over time, suggesting that repeated practice is necessary for long-term retention [54].

For duodenal atresia models, animal-based simulations, such as those by Ordorica-Flores et al. and Etlinger et al. (2022), demonstrated strong face and content validity [57, 59]. In contrast, inanimate models such as those developed by Barsness et al. provided a feasible, non-animal alternative but required further validation to confirm skill transferability [58].

Studies evaluating laparoscopic fundoplication models suggest that structured training programs improve pediatrics surgeons' technical skills. Jimbo et al. demonstrated that endoscopic training enhanced instrument control and precision, particularly in bi-hand coordination [63, 102]. However, while their laparoscopic simulator differentiated general surgeons from pediatrics surgeons, its long-term impact on surgical competency remains unclear. Similarly, Onishi et al. observed that motion-tracking in virtual simulation revealed variations in technique, suggesting that a combination of physical and virtual training may be optimal [64].

These findings demonstrate that 3D-printed hepatobiliary models, as described by Burdall et al. provide a cost-effective alternative for laparoscopic choledochal surgery training. In contrast, inanimate models using animal tissue, such as those developed by Jimbo et al., offer improved realism but face challenges related to accessibility and reproducibility [66, 68]. Based on our findings, combining 3D-printing technology with anatomical accuracy from animal-based models could enhance hepatobiliary surgical training programs.

For thoracic surgery training, the results showed that synthetic and rapid prototyped models, such as those by Barsness et al., Takazawa et al., and Harada et al. effectively differentiated skill levels through OSATS scoring and motion-tracking analysis [48, 71, 72]. On the other hand, animal-based models, including those by Marecos et al. and Kirlum et al. demonstrated high anatomical fidelity but at the same time raised ethical and logistical concerns [73, 74]. Overall, these connections highlight the importance of integrating motion-tracking, quantitative skill assessment, and comparative validation studies into pediatrics thoracic surgical training. Relevant studies favor 3D-printed synthetic models for laparoscopic pyeloplasty training, with studies by Krois et al. demonstrating strong feasibility and high user acceptance [80]. Therefore, patient-specific 3D-printed models could play a crucial role in preoperative training, particularly for complex pediatrics urological procedures.

## Conclusion

This systematic review underlines the importance of standardized, validated training models for newborn/infant MIS. While significant advancements have been made, further comparative studies are necessary to determine the optimal simulation models for structured pediatric surgical education. Future research should focus on refining synthetic models to enhance anatomical accuracy and sustainability while integrating validated proficiency-based curricula into training programs. Future research should focus on developing standardized newborn/infant MIS training programs incorporating validated simulation models, establish objective performance metrics for evaluating surgical proficiency in neonatal MIS, expand the availability of high-fidelity, cost-effective synthetic models to reduce reliance on animal-based training, and investigate long-term skill retention and patient outcomes associated with simulation-based training interventions*.*

## Limitations

This systematic review has several limitations that should be acknowledged. First, although a comprehensive literature search was conducted across multiple databases following PRISMA guidelines, the inclusion was limited to English language publications, which may have introduced language bias and excluded relevant studies published in other languages. Second, the heterogeneity among included studies in terms of study design, validation methods, simulator types, participant demographics, and outcome measures posed a challenge in synthesizing an analysis of findings. Due to this variation, direct comparisons between models were often not feasible, and meta-analysis could not be performed. Furthermore, while many models reported content and construct validity, only a minority utilized objective, standardized assessment tools such as OSATS or motion-tracking systems to validate performance outcomes, thereby limiting the robustness of evidence. Long-term impacts of simulation-based training—such as retention of skills, transferability to clinical practice, or effects on patient outcomes—were rarely assessed. This is particularly relevant when evaluating the true educational value of these simulators in pediatric MIS. Moreover, while efforts were made to categorize the models according to LoE and LoR, these classifications were sometimes based on author-reported data or subjective interpretation as formal grading systems were not uniformly applied across studies. Lastly, the rapidly evolving field of 3D printing and simulation technologies means that some emerging models may not yet be captured in published literature, and new advancements could quickly outpace the findings of this review.

## Data Availability

No datasets were generated or analysed during the current study.
